# Immune Activation, Immunosenescence, and Osteoprotegerin as Markers of Endothelial Dysfunction in Subclinical HIV-Associated Atherosclerosis

**DOI:** 10.1155/2014/192594

**Published:** 2014-10-14

**Authors:** Alessandra D'Abramo, Maria Antonella Zingaropoli, Alessandra Oliva, Claudia D'Agostino, Samir Al Moghazi, Giulia De Luca, Marco Iannetta, Claudio Maria Mastroianni, Vincenzo Vullo

**Affiliations:** Department of Public Health and Infectious Diseases, “ Sapienza” University of Rome, Viale del Policlinico 155, 00161 Rome, Italy

## Abstract

HIV-infected patients have a significantly greater risk of cardiovascular disease. Several markers including osteoprotegerin have been shown to be involved in the development and progression of atherosclerosis. We investigated the relationship between T-cell phenotype, osteoprotegerin, and atherosclerosis evaluated by carotid intima-media thickness (c-IMT) in 94 HIV+ patients on suppressive antiretroviral therapy with Framingham score <10%. As for the control group, 24 HIV-negative subjects were enrolled. c-IMT was assessed by ultrasound. CD4+/CD8+ T-cell activation (CD38+ HLADR+) and senescence (CD57+ CD28−) were measured by flow cytometry. IL-6 and OPG levels were measured by ELISA kit. c-IMT was higher in HIV+ than in controls. Among HIV+ patients, 44.7% had pathological c-IMT (≥0.9 mm). CD8+ T-cell activation and senescence and OPG plasma levels were higher in HIV+ patients than in controls. Subjects with pathological c-IMT exhibited higher CD8+ immune activation and immunosenescence and OPG levels than subjects with normal c-IMT. Multivariate analysis showed that age, CD8+ CD38+ HLADR+, and CD8+ CD28− CD57+ were independently associated with pathological c-IMT. Several factors have been implicated in the pathogenesis of atherosclerosis in HIV patients. Immune activation and immunosenescence of CD8+ T cell together with OPG plasma levels might be associated with the development and progression of early atherosclerosis, even in the case of viral suppression.

## 1. Introduction

HIV-infected patients, with the increasing life expectancy, appear to have a significantly greater risk of cardiovascular disease (CVD) than HIV-negative individuals. It now appears clear that both HIV infection itself and antiretroviral therapy (ART) are associated with a higher risk of CVD and metabolic disorders. CVD, which occurs in the general population with advancing age, seems to be correlated with a premature aging in HIV-infected patients, occurring at an earlier age in HIV-infected than in uninfected subjects [1, 2]. Moreover, recent studies showed how the risk is elevated also in HIV-positive subjects virologically suppressed and with a low Framingham score [3]. An important aspect of this premature aging is the immune activation and the consequent immunosenescence that causes a thymic involution, a reduced circulating naive T cells, an increased number of CD4+ well-differentiated CD28− T cells, and an increased level of proinflammatory cytokines [4–7]. Recent studies have introduced the hypothesis that chronic inflammation, immune activation, and immunosenescence might contribute to the endothelial activation/dysfunction with consequent atherosclerosis in the setting of HIV infection [8]. Several markers, such as VCAM-1, ICAM-1, and von Willebrand factor antigen, have been shown to reliably indicate the increased activation of endothelial cells in atherosclerosis. Tumor necrosis factor-*α* (TNF-*α*) has been implicated in myocardial dysfunction resulting from acute coronary syndrome and high levels of C-reactive protein and IL-1 and IL-6 have been associated with subclinical atherosclerosis [9–12]. Furthermore, new soluble markers including osteoprotegerin (OPG), member of the TNF superfamily, have been shown to be involved in the development and progression of atherosclerosis [13, 14]. Given the morbidity and the mortality associated with overt CVD, early identification and management of subclinical disease are desirable before complications of overt CVD develop [15]. Measurement of carotid intima-media thickness (c-IMT) has previously been correlated with the extent of coronary atherosclerosis and increases in c-IMT are predictive of future CVD events [16]. The aim of our study was to investigate the relationship of lymphocytes T-cell phenotype, IL-6, and OPG plasma levels with atherosclerosis evaluated by c-IMT in HIV-positive patients on suppressive ART.

## 2. Materials and Methods

### 2.1. Ethics Statement

The study protocol designed according to the Helsinki Declaration II was approved by the local ethics committee. All the patients gave written informed consent to participate.

### 2.2. Patients

We recruited 118 patients from the Department of Public Health and Infectious Diseases of “Sapienza” University of Rome. Ninety-four were HIV-infected subjects on ART since 48 weeks with undetectable viremia (<37 copies/mL) and low cardiovascular diseases risk defined by a Framingham score <10%. As for the control group, we enrolled 24 HIV-negative individuals matched for age, gender, and Framingham score. For each patient, we collected medical and family history, lifestyle, smoking status, ART, HIV-RNA zenith, and nadir CD4+ cell count. Current lymphocytes T CD4+ and CD8+ cell count was determined by flow cytometric analysis (MACSQuant Analyzer, Miltenyi Biotec, Germany) and HIV-1 RNA plasma levels were detected by a quantitative reverse polymerase chain reaction (Amplicor HIV Monitor; Roche Diagnostic System, Branchburg, NJ, version 1.5, l.o.d. 37 copies/mL). Triglycerides, total cholesterol, high density lipoprotein cholesterol (HDL), and low density lipoprotein cholesterol (LDL) were measured in blood samples. Body mass index (BMI) was calculated (kg/m^2^) and recorded for each individual. Due to the high influence of CMV on immunosenescence and due to immune activation and in order to eliminate the CMV as a confounding factor, only CMV positive subjects were included in both study populations. Exclusion criteria were age <18 years, previous virological failure, recent AIDS-defining illness, coinfection with hepatitis virus, and presence of other comorbidities (metabolic syndrome, diabetes mellitus, arterial hypertension, kidney disease, and hormonal dysfunction).

### 2.3. IL-6 and Osteoprotegerin Detection

IL-6 was measured with an ELISA kit (eBioscience Bender MedSystems, Inc., Vienna, Austria). The detection limit of assay was 3.1 pg/mL. Osteoprotegerin (OPG) levels were measured by ELISA kits (Biomedica Gruppe, Vienna, Austria). The detection limit of assay was 0.12 pmol/L.

### 2.4. Immune Activation and Immunosenescence

Lymphocyte surface phenotypes were evaluated by flow cytometry using fresh peripheral blood. For the activation and the senescence analysis of CD4+ and CD8+ T cells, the following fluorochrome-labeled antibodies were used: Pacific Blue-CD3 (BioLegend, 500 uL), FITC-CD28 (BioLegend, 2 mL), PE-CD57 (BioLegend, 2 mL), PerCp/Cy5.5-HLA DR (BioLegend, 500 uL), Pe/Cy7-CD8 (BioLegend, 2 mL), CD38-APC (BioLegend, 2 mL), and APC/Cy7-CD4 (BioLegend, 2 mL). The flow cytometer was calibrated using MACSQuantTM Calibration Beads and for the automatic compensation marked beads (BDTM CompBeads anti-mouse Ig, k) and unmarked beads (BDTM CompBeads negative control, FBS) were used. For the lysis of red blood cells, FACS Lysing Solution (BD, Lysing Solution) was used. The flow cytometer used for the analysis was the MACSQuant Analyzer with 8 channels and the data were analyzed using the FlowJo V10 software. Before each acquisition, both calibration and automatic compensation were performed. Immune activation was defined as HLADR+ CD38+ whereas immunosenescence was defined as CD57+ CD28− [17, 18].

### 2.5. Carotid Intima-Media Thickness Measurement

c-IMT measurement was obtained for each patient using a B-mode ultrasound recording with a 7 to 14 MZ array probe (Esaote Technology). Patients must lay in supine position in a dark room with a slight hyperextension and must turn the neck to the opposite side. The common carotid, the bifurcation, and at least the first 2 cm of the internal carotid were examined on the long and short axes. In addition, 3 measurements were made at the far and near walls of each internal carotid and specifically at the carotid bifurcation and the bulb and 1 cm after the bifurcation. The mean value (expressed as mm) of the 3 measurements taken at each site of the internal carotid (left and right) was calculated for each patient and used as the final measurement of internal c-IMT. According to published population studies, we defined normal c-IMT as IMT <0.9 mm and pathological c-IMT as IMT >0.9 mm [19]. In order to avoid interoperator differences, all the measurements of c-IMT were performed by a single operator.

### 2.6. Statistical Analysis

Continuous data were analyzed with Student's *t* test, whereas the nonparametric Mann-Whitney test was applied for values not normally distributed. Pearson correlation coefficient was used for correlations. Linear regression model was tested to evaluate the association between lymphocytes T-cell phenotype, IL-6, OPG, and c-IMT. To explore the factors independently associated with c-IMT, multivariable logistic regression was performed. Data were expressed as median (range) or mean ± standard deviation (SD), as appropriate. A *P* value of <0.05 was considered statistically significant. Statistical analyses were performed using STATA (version 9) software (STATA Corp. LP, College Station, TX, USA).

## 3. Results

### 3.1. Characteristics of the Study Population

Out of 94 HIV-infected subjects with a low cardiovascular diseases risk (Framingham score <10%), 72 (76.5%) were males and 22 (23.5%) were females with a mean age of 47.4 ± 11.4 years. 52.1% were not smokers and mean BMI was 20.7 ± 2.4 kg/m^2^. Median nadir and current lymphocytes T CD4+ cells count were 195.5 cell/mmc (range 4–1318) and 643.5 cell/mmc (range 159–1705), respectively. Out of 94 HIV-infected subjects on ART, 62.7% were on PI and 37.3% were on NNRTI based regimen. All the patients were CMV positive. The mean plasma concentration of total cholesterol was 188.3 ± 47.4 mg/dL, HDL cholesterol was equal to 48 ± 14.6 mg/dL, LDL cholesterol was equal to 114 ± 46.3 mg/dL, and triglycerides were equal to 145.3 ± 80.9 mg/dL. None of the subjects were receiving lipid-lowering therapy. General characteristics of study population are summarized in Table 1.

### 3.2. Carotid Intima-Media Thickness

c-IMT was higher in HIV+ than in controls (mean ± SD: 0.85 ± 0.17 versus 0.28 ± 0.24 mm; median values: 0.835 versus 0.475 mm, *P* < 0.001). Among HIV+ patients, 52/94 (55.3%) demonstrated a normal c-IMT, whereas 42/94 (44.7%) had a pathological c-IMT.

### 3.3. Immune Activation

**Figure 1:**
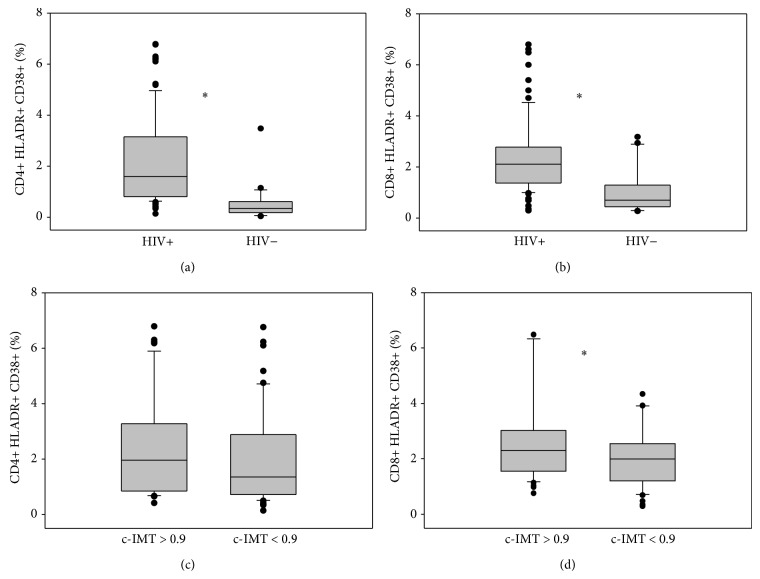
CD4+ (a) and CD8+ (b) immune activation in HIV-infected subjects compared with HIV-negative controls. CD4+ (c) and CD8+ (d) immune activation in HIV-infected subjects according to c-IMT. Values are expressed as percentage. Horizontal bars represent median. Upper and lower whisker mean third quartile +1.5 (interquartile range (IQR)) and first quartile-1.5(IQR).  ^*^It represents a *P* value < 0.05.

In comparison to HIV-negative patients, HIV-positive subjects exhibited higher levels of lymphocytes T CD4+ expressing HLADR+ CD38+ (median values 1.6% versus 0.34%, *P* < 0.001) and CD8+ HLADR+ CD38+ (median values 2.1% versus 0.69%, *P* < 0.001). Patients with pathological c-IMT had higher expression of lymphocytes T CD4+ and CD8+ HLADR+ CD38+ (median values 1.96% versus 1.35%, *P* = 0.124; median values 2.27% versus 1.99%, *P* = 0.048, resp.) than patients with normal c-IMT (Figure 1).

### 3.4. Immunosenescence

**Figure 2:**
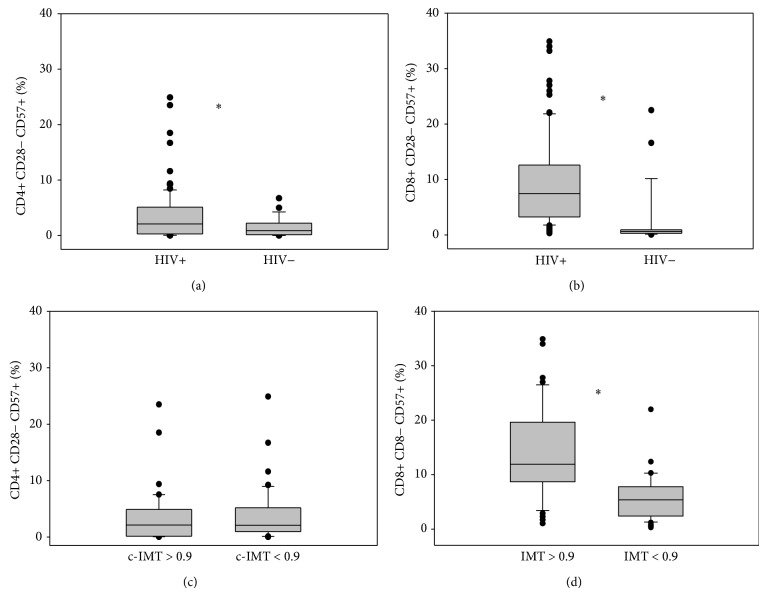
CD4+ (a) and CD8+ (b) immunosenescence in HIV-infected subjects compared with HIV-negative controls. CD4+ (c) and CD8+ (d) immunosenescence in HIV-infected subjects according to c-IMT. Values are expressed as percentage. Horizontal bars represent median. Upper and lower whisker mean third quartile +1.5 (interquartile range (IQR)) and first quartile-1.5(IQR).  ^*^It represents a *P* value < 0.05.

HIV-positive subjects exhibited a higher level of lymphocytes T CD4+ and CD8+ CD57+ CD28− (median values 2.08% versus 0.87%, *P* = 0.026; median values 7.44% versus 0.62%, *P* < 0.001, resp.) compared with HIV-negative patients. Patients with pathological c-IMT had higher expression of lymphocytes T CD4+ and CD8+ CD57+ CD28− (median values 2.1% versus 2%, *P* = 0.442; median values 11.9% versus 4.8%, *P* < 0.001, resp.) than patients with normal c-IMT (Figure 2). In linear regression analysis, there was a positive correlation between lymphocytes T CD8+ HLADR+ CD38+ and CD8+ CD57+ CD28− (*P* = 0.002). Moreover, there was a negative correlation between lymphocytes T CD4+ nadir and lymphocytes T CD4+ and T CD8+ HLADR+ CD38+ (*P* = 0.007 and *P* = 0.087, resp.). The same trend was observed for lymphocytes T CD4+ nadir and lymphocytes T CD4+ CD57+ CD28− and CD8+ CD57+ CD28−.

### 3.5. Osteoprotegerin and Interleukin-6

**Figure 3:**
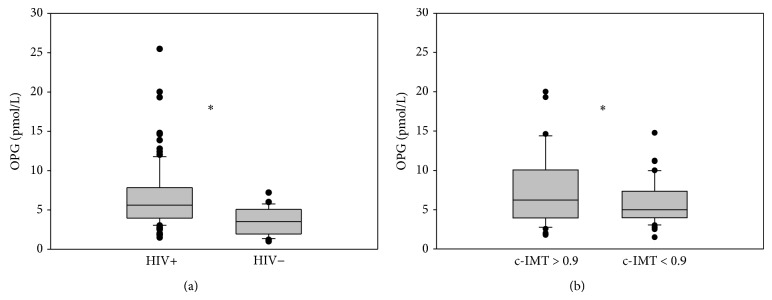
OPG plasma levels in HIV-infected subjects compared with HIV-negative controls (a) and in HIV-infected subjects according to c-IMT (b). Values are expressed as pmol/L. Horizontal bars represent median. Upper and lower whisker mean third quartile +1.5 (interquartile range (IQR)) and first quartile-1.5(IQR).  ^*^It represents a *P* value < 0.05.

OPG plasma levels were significantly higher in HIV-infected patients than in healthy controls (mean ± SD: 6.59 ± 4 pmol/L versus 3.57 ± 1.6 pmol/L; median: 5.6 versus 3.5 pmol/L) (*P* < 0.001) (Figure 3(a)). IL-6 plasma levels were higher in HIV-positive subjects than in HIV-negative individuals (mean ± SD 49.6 ± 29.4 versus 37.4 ± 8 pg/mL; median: 44.3 versus 36.5 pg/mL) (*P* = 0.096). Patients with pathological c-IMT had higher OPG plasma levels (median values 6.22 pmol/L versus 4.86 pmol/L, *P* = 0.05) (Figure 3(b)) and IL-6 plasma levels (median values 46 pg/mL versus 40.7 pg/mL, *P* = 0.156) than patients with normal c-IMT. Moreover, lymphocytes T CD8+ HLADR+ CD38+ positively correlated with OPG plasma level (*P* = 0.008). Although not statistically significant, lymphocytes T CD4+ HLADR+ CD38+, CD4+ CD57+ CD28− and CD8+ CD57+ CD28− were associated with higher OPG and IL-6 plasma levels.

### 3.6. Univariate and Multivariate Analysis

The univariate model revealed a significant association between c-IMT and age (*P* < 0.001), time of diagnosis (*P* = 0.055), lymphocytes T CD8+ CD57+ CD28− (*P* < 0.001), lymphocytes T CD4+ HLADR+ CD38+ (*P* = 0.084), and an inverse correlation between c-IMT and lymphocytes T CD4+ nadir (*P* = 0.008). Moreover, HIV+ subjects with pathological c-IMT had lymphocytes T CD4+ nadir lower than HIV+ subjects with normal c-IMT (*P* = 0.032). However, when these data were analyzed by multivariable logistic regression, only age (OR = 1.008; confidence interval CI = 1.005–1.011; *P* < 0.001), PI exposure (OR = 1.050; confidence interval CI = 0.992–1.111; *P* = 0.009), lymphocytes T CD8+ HLADR+ CD38+ (OR = 1.234; CI = 1.006–1.042; *P* = 0.011), and lymphocytes T CD8+ CD57+ CD28− (OR = 1.008; CI = 1.004–1.011; *P* < 0.001) were confirmed to be independently associated with c-IMT (Table 2).

## 4. Discussion

Cardiovascular disease, as already known, occurs at an earlier age in HIV-infected than in uninfected subjects [1, 20]. An important aspect of this premature aging is the immune activation and the consequent immunosenescence that causes a thymic involution, a reduced circulating naive T-cells, an increased number of CD4+ well-differentiated CD28− T cells, and an increased level of proinflammatory cytokines (IL-6, TNF*α*) [4, 18, 21–25]. Recent studies have introduced the hypothesis that chronic inflammation and immune activation can contribute to the initiation and progression of atherosclerosis in the setting of HIV infection [26–30]. Moreover, it has been widely described that ART is implicated in the atherosclerosis process, and, in particular PI based regimens [31]. In this study, we evaluated the relationship between lymphocytes T-cell phenotype and IL-6 and OPG plasma levels and c-IMT in HIV-positive patients on ART. We enrolled 118 patients, 94 HIV-infected subjects on ART since 48 weeks with undetectable viremia (<37 copies/mL) and low cardiovascular diseases risk defined by a Framingham score <10% and 24 HIV-negative individuals matched for age, gender, and Framingham score. We observed an increased c-IMT in HIV-positive patients than in healthy controls and, among HIV+ patients, 42/94 (44.7%) had pathological c-IMT (≥0.9 mm). Several studies showed that the measurement of c-IMT has been correlated with the extent of coronary atherosclerosis and that the increase of c-IMT is predictive of future CVD events. c-IMT is strongly associated with the traditional risk factors such as male sex, ageing, overweight, high blood cholesterol, diabetes and insulin resistance, smoking, and, in particular, elevated blood pressure, probably due to media hypertrophy [16]. New or emerging risk factors such as various lipoproteins, psychosocial status, plasma viscosity, and hyperhomocysteinemia have also been associated with c-IMT values. In the setting of HIV infection, additional factors play a role in the pathogenesis and progression of atherosclerosis where HIV itself is one of the major players. HIV determines a state of chronic inflammation with activation and progressive aging of the immune system [4, 23–25]. Recently, some authors have also suggested an association between T-cell activation/senescence and markers of subclinical carotid artery disease, even among patients on stable ART [9, 30]. In this paper, we observed a higher level of activated and senescent CD4+ and CD8+ lymphocytes T in HIV-positive subjects than in general population, although ART provides full suppression of HIV viremia. Moreover, HIV subjects with pathological c-IMT showed levels of immune activation and immunosenescence higher than HIV-subjects with normal c-IMT. The role of inflammation and endothelial activation/dysfunction in the development of atherosclerosis has been extensively studied in the general population and several markers, such as VCAM-1, ICAM-1, and von Willebrand factor antigen, have been shown to reliably indicate the increased activation of endothelial cells in atherosclerosis. TNF-*α* has been implicated in myocardial dysfunction resulting from acute coronary syndrome and high levels of IL-1 and IL-6 have been associated with subclinical atherosclerosis [9, 10]. Furthermore, new soluble markers including OPG have been shown to be involved in the development and progression of atherosclerosis [32–34]. The OPG/RANK/RANKL system, member of TNF superfamily and mostly implicated in bone remodelling, is involved in immune and in vascular system [35–38]. In fact, RANKL, which is expressed by osteoblast cells and their precursor, activates its receptor (RANK), expressed by osteoclast cells and their precursor, thus promoting osteoclast formation, activation, and prolonging osteoclast survival. The effects of RANKL are blocked by the secretory glycoprotein OPG, which acts as a decoy receptor for RANKL. Changes in the RANKL/OPG ratio are critical in the pathogenesis of bone disease. The relationship between bone and vascular disease is known; in this contest, OPG could be considered as a bridge from bone to vascular system. The role of OPG in cardiovascular disease is still debated. OPG might contribute to endothelial dysfunction by blocking RANKL signalling which is able to activate protective intracellular endothelial pathways such as the nitric oxide synthase pathway, to increase the adhesion and migration of inflammatory cells through the endothelium and the activity of metalloproteases [32, 39, 40]. In a previous study, we found higher OPG plasma levels in HIV-positive subjects than in healthy controls, suggesting an association between OPG plasma levels and cardiovascular disease. The increased OPG plasma concentration found in HIV-positive patients with low cardiovascular risk may suggest that OPG is implicated in the early phase of atherosclerosis development process. We showed that OPG plasma concentrations are associated with atherosclerosis in HIV-infected subjects with a low Framingham score. Therefore, OPG plasma measurement could be a useful and noninvasive tool in clinical practice in order to early discriminate subjects at risk of developing atherosclerosis [13]. Although in the multivariate analysis OPG has not been found to be an independent factor associated with c-IMT, OPG plasma levels were significantly higher in HIV-infected patients than in healthy controls (*P* < 0.001) and patients with pathological c-IMT had higher OPG plasma levels than HIV-positive subjects with normal c-IMT (*P* = 0.05). Moreover, OPG plasma levels were strictly correlated with lymphocytes T CD8+ HLADR+ CD38+, supporting the relationship with immune activation, cytokine production, and atherosclerosis. Thus, our hypothesis is that the high levels of immune activation and immunosenescence of CD8 T cells might influence the production of OPG leading to increased c-IMT in HIV-positive subjects. The measurement of OPG plasma levels together with CD8 T cells immune activation and immunosenescence might be a useful and noninvasive parameter in order to identify early atherosclerosis in HIV-positive subjects with low cardiovascular risk.

## 5. Conclusions

Several factors have been implicated in the pathogenesis of atherosclerosis in HIV patients. Age, PI exposure, and lymphocytes T CD8+ HLADR+ CD38+ and T CD8+ CD57+ CD28− were confirmed to be independently associated with c-IMT. In conclusion, the immune activation and immunosenescence of CD8+ T cells together with OPG plasma levels might be associated with the development and progression of early atherosclerosis in HIV-infected patients, even in the case of viral suppression.

## Supplementary Material

The linear regression analyses showed that c-IMT positively correlated with age, time of diagnosis, immuneactivated and immunosenescent CD8+ T cells and negatively correlated with CD4+ nadir T cells. Immuneactivated CD8+ T cells positively correlated with immunosenescent CD8+ T cells and OPG plasma levels. CD4+ nadir T cells negatively correlated with immuneactivated CD4+ and CD8+ T cells.

## Figures and Tables

**Table 1:** Clinical characteristics of study population.

	HIV+	HIV−	*P* value
Age (M ± SD)	47.4 ± 11.4	48 ± 9	*P = 0.432 *
Sex	72 M, 22 F	18 M, 6 F	*P = 0.581 *
Smoke status (*n*, %)			
No	49 (52.1%)	15 (62.5%)	*P = 0.745 *
Yes	45 (47.9%)	9 (37.5%)	*P = 0.669 *
CD4+ (mmc) (M ± SD)	685.8 ± 395.5	770 ± 221	*P = 0.485 *
CD4+ % (M ± SD)	27.3 ± 7.6	36 ± 9.3	*P = 0.563 *
CD4+ (mmc) nadir (M ± SD)	242.3 ± 227.6	—	n.a.
CD4+ % nadir (M ± SD)	14.7 ± 9.6	—	n.a.
HIV-RNA zenith (cp/mL) (M ± SD)	297218.1 ± 114812.4	—	n.a.
HIV-RNA (cp/mL)	<37	<37	n.a.
ART (*n*, %)	94/94 (100%)	—	n.a.
PI based regimen	59/94 (62.7%)		
NNRTI based regimen	35/94 (37.3%)		
CMV serostatus (*n*, %)	94/94 (100%)	24/24 (100%)	n.a.
Lipid-lowering therapy			
No	94 (100%)	24 (100%)	n.a
Yes	0	0	
SBP (mmHg)	120.3 ± 12.5	116.8 ± 18.4	*P = 0.362 *
DBP (mmHg)	80.2 ± 10.6	83.5 ± 12.1	*P = 0.175 *
Triglycerides (mg/dL) (M ± SD)	145.3 ± 80.9	109.6 ± 42	*P = 0.921 *
Cholesterol total (mg/dL) (M ± SD)	188.3 ± 47.4	144.3 ± 37.4	*P = 0.875 *
Cholesterol HDL (mg/dL) (M ± SD)	48 ± 14.6	53.2 ± 16.6	*P = 0.396 *
Cholesterol LDL (mg/dL) (M ± SD)	114 ± 46.3	102.5 ± 22.4	*P = 0.581 *
Body mass index (kg/m^2^)	21 ± 2.2	19 ± 2.7	*P = 0.497 *
Framingham score (%)	7.1 ± 2.8	3.2 ± 2.1	*P = 0.694 *

M: mean; SD: standard deviation; ART: antiretroviral therapy; PI: protease inhibitor; NNRTI: nonnucleoside reverse-transcriptase inhibitor; CMV: cytomegalovirus; SBP: cystolic blood pressure; DBP: diastolic blood pressure; HDL: high density lipoprotein; LDL: low density lipoprotein.

**Table 2:** Multivariate analysis.

	OR	95% CI	*P* value
Age	1.008	1.005–1.011	***<0.001***
Sex	1.045	0.980–1.115	*0.185 *
Smoke	1.002	0.947–1.060	*0.942 *
Time of diagnosis	1.002	0.998–1.005	*0.305 *
PI exposure	1.050	0.992–1.111	***0.009***
Cholesterol total	1.000	0.999–1.003	*0.934 *
Cholesterol HDL	1.000	0.997–1.003	*0.952 *
Cholesterol LDL	1.000	0.999–1.001	*0.651 *
Triglycerides	0.999	0.999-1.000	*0.172 *
OPG	0.998	0.990–1.006	*0.632 *
IL-6	1.000	0.999–1.001	*0.744 *
CD4+ nadir	0.999	0.999-1.000	*0.264 *
CD4+ current	1.000	0.998–1.001	*0.478 *
CD4+ HLADR+ CD38+	1.004	0.985–1.023	*0.692 *
CD8+ HLADR+ CD38+	1.234	1.006–1.042	***0.011***
CD4+ CD28− CD57+	0.993	0.987–1.000	*0.063 *
CD8+ CD28− CD57+	1.008	1.004–1.011	***<0.001***

OR: odd ratio; CI: confidence interval; PI: protease inhibitor; HDL: high density lipoprotein; LDL: low density lipoprotein OPG: osteoprotegerin; IL-6: interleukin-6.
