# Supplementary material for: Immune Activation, Immunosenescence, and Osteoprotegerin as Markers of Endothelial Dysfunction in Subclinical HIV-Associated Atherosclerosis
Source: Mediators Inflamm. 2014 Oct 14;2014:192594. doi: 10.1155/2014/192594 (PMC4211147; doi:10.1155/2014/192594)
Supplement: Supplementary file 1 — The linear regression analyses showed that c-IMT positively correlated with age, time of diagnosis, immuneactivated and immunosenescent CD8+ T cells and negatively correlated with CD4+ nadir T cells. Immuneactivated CD8+ T cells positively correlated with immunosenescent CD8+ T cells and OPG plasma levels. CD4+ nadir T cells negatively correlated with immuneactivated CD4+ and CD8+ T cells. [file 192594.f1.docx]

**Supplementary Table** . Linear regression analysis in the HIV infected subjects.

| **POSITIVE CORRELATIONS** | ***p-value*** |
| --- | --- |
| **c-IMT** |  |
| Age | *p <0.001* |
| Time of Diagnosis | *p=0.055* |
| CD8+ CD57+ CD28- T-cells | *p <0.001* |
| CD4+ HLADR+ CD38+ T-cells | *p=0.084* |
| **CD8+ HLADR+ CD38+ T-cells** |  |
| CD8+ CD57+ CD28- T-cells | *p=0.002* |
| OPG | *p=0.008* |
|  |  |
| **NEGATIVE CORRELATIONS** | ***p-value*** |
| **c-IMT** |  |
| CD4+ nadir | *p=0.008* |
| **CD4+ nadir T-cells** |  |
| CD4+ HLADR+ CD38+ T-cells | *p=0.007* |
| CD8+ HLADR+ CD38+ T-cells | *p=0.087* |
